# *ARLTS1* and Prostate Cancer Risk - Analysis of Expression and Regulation

**DOI:** 10.1371/journal.pone.0072040

**Published:** 2013-08-05

**Authors:** Sanna Siltanen, Daniel Fischer, Tommi Rantapero, Virpi Laitinen, John Patrick Mpindi, Olli Kallioniemi, Tiina Wahlfors, Johanna Schleutker

**Affiliations:** 1 Institute of Biomedical Technology/BioMediTech, University of Tampere and Fimlab Laboratories, Tampere, Finland; 2 School of Health Sciences, University of Tampere, Tampere, Finland; 3 Institute for Molecular Medicine (FIMM), University of Helsinki, Helsinki, Finland; 4 Department of Medical Biochemistry and Genetics, Institute of Biomedicine, University of Turku, Turku, Finland; Ohio State University Medical Center, United States of America

## Abstract

Prostate cancer (PCa) is a heterogeneous trait for which several susceptibility loci have been implicated by genome-wide linkage and association studies. The genomic region 13q14 is frequently deleted in tumour tissues of both sporadic and familial PCa patients and is consequently recognised as a possible locus of tumour suppressor gene(s). Deletions of this region have been found in many other cancers. Recently, we showed that homozygous carriers for the T442C variant of the *ARLTS1* gene (ADP-ribosylation factor-like tumour suppressor protein 1 or *ARL11*, located at 13q14) are associated with an increased risk for both unselected and familial PCa. Furthermore, the variant T442C was observed in greater frequency among malignant tissue samples, PCa cell lines and xenografts, supporting its role in PCa tumourigenesis. In this study, 84 PCa cases and 15 controls were analysed for *ARLTS1* expression status in blood-derived RNA. A statistically significant (p = 0.0037) decrease of *ARLTS1* expression in PCa cases was detected. Regulation of *ARLTS1* expression was analysed with eQTL (expression quantitative trait loci) methods. Altogether fourteen significant *cis*-eQTLs affecting the *ARLTS1* expression level were found. In addition, epistatic interactions of *ARLTS1* genomic variants with genes involved in immune system processes were predicted with the MDR program. In conclusion, this study further supports the role of *ARLTS1* as a tumour suppressor gene and reveals that the expression is regulated through variants localised in regulatory regions.

## Introduction

Prostate cancer (PCa) is a heterogeneous trait, and it is the most common malignancy among men in western countries, including Finland. It is a multifactorial disease, and definitive risk factors include age, ethnic origin and family history. In Finland, the incidence of PCa is 89.4/100,000, and in 2010, 4697 new prostate cancer cases were diagnosed (http://www.cancer.fi/syoparekisteri/en/). Despite extensive research over the last decade, the etiological risk factors and genes that cause genetic susceptibility remain largely unknown. This lack of knowledge has hampered effective cancer prevention and development of better treatments.

Mutations in the known high-penetrance PCa predisposition genes explain only a small fraction of PCa cases. A polygenic model for familial aggregation of cancer has been proposed where several low-penetrance alleles may have a multiplicative and/or modifying effect. One low-penetrant candidate gene is *ARLTS1* (*ARL11*), ADP-ribosylation factor like tumour suppressor protein 1, a putative tumour-suppressor gene on chromosome 13q14, which has been shown to function in many human cancers [1]–[5]. *ARLTS1* is a member of the ADP-ribosylation factor family that plays a role in apoptotic signalling. The same chromosomal area on 13q has been indicated in a multi-centre genome-wide linkage study in families with at least five affected members [6]. In another recent study, the immediate adjacent region 13q13 showed a suggestive linkage to PCa, with a HLOD>1.9 [7]. In addition, the locus 13q14 is among the most frequently deleted chromosomal regions in somatic tumour tissues in both unselected and hereditary prostate cancers [8], [9], suggesting that *ARLTS1* could be a target for both germline and somatic mutations. We previously reported a significant association of *ARLTS1* T442C (rs3803185) homozygote carriers with PCa [10]. This risk genotype was also associated with decreased *ARLTS1* expression in the lymphoblastoid cell line samples of PCa patients, and *ARLTS1* co-expression signatures from data mining revealed that *ARLTS1* expression was strongly associated with immune system processes. These processes are of interest because a link between chronic inflammation and PCa progression has been repeatedly proposed, and multiple genes acting in inflammatory pathways have been linked to PCa susceptibility [11]–[13].

Complex diseases, such as cancer, are caused by a combination of multiple genetic interactions and environmental factors, which are hard to detect and link to each other. To determine true causal associations using statistical methods and phenotype information, it is advisable to organise individual markers into groups according to meaningful biological criteria, such as inflammation. By composing variant sets, it is possible to reduce the number of hypotheses being tested, which allows the association between a genomic feature and a phenotype to be more easily detected.

Epistasis in genotype level is defined as the interaction among multiple genes or loci, and this joint genetic effect may be the factor behind “missing heritability”, a phenomenon linked to the unexplained portion of hereditary cancer susceptibility, which is observed in PCa. The genome-wide expression quantitative trait loci, eQTL, analysis is a method for studying epistasis in complex traits that is able to detect associations between genotypic and expression data. The eQTL analysis is a widely used approach to gain insight into the role of single nucleotide polymorphisms (SNPs) affecting transcript levels.

In this study, we investigated the mechanisms behind the previously observed association of *ARLTS1* and PCa by focusing on finding gene/expression interactions that dispose patients to PCa, including interactions involving the *ARLTS1* gene. To further investigate the *ARLTS1* expression differences seen previously in tumor samples, prostate cancer and lymphoblastoid cell lines [10], we performed functional eQTL analysis from whole blood derived total RNA from PCa patients. The previously reported *ARLTS1* co-expression with immune system processes [10] was tested by MDR analysis. To our knowledge, this is the first study reporting the findings of *ARLTS1* interacting variants and prostate cancer eQTLs at the 13q14 region.

## Materials and Methods

### Study population

All the samples were of Finnish origin. The identification and collection of the Finnish HPC families has been described elsewhere [14]. The familial samples analysed in this study had at least two affected first or second degree relatives. Altogether, 102 prostate cancer cases and 33 healthy male family members belonging to 31 families were initially taken into the study population. The clinical characteristics of the familial patients used in RNA sequencing (n = 84) are referred to in Table 1. Average age at diagnosis was 63.0 y.

**Table 1:** Clinicopathologic findings at diagnosis of the PCa patients used in RNA sequencing (n = 84).

	*n (%)*
Age at diagnosis	
<65 years	48 (57.1)
≥65 years	35 (41.7)
Stage	
T stage	
T1 (clinically undetectable)	31 (36.9)
T2–T4 (clinically detectable)	48 (57.1)
M stage	
M0 (no evidence of metastasis)	53 (63.1)
M1 (bone metastasis)	0 (0)
MX (bone metastasis cannot be assessed)	27 (32.1)
PSA value	
<20 ng/ml	60 (71.4)
≥20 ng/ml	14 (16.7)
Grade	
Gleason score	
<7	44 (52.4)
7	10 (11.9)
>7	0 (0)

Patient information and samples were obtained with full written informed consent. The study was performed under appropriate research permissions from the Ethics Committees of the Tampere University Hospital, Finland, as well as the Ministry of Social Affairs and Health in Finland.

### Genome-wide SNP Genotyping

Genome-wide SNP genotyping was performed using the HumanOmniExpress BeadChip microarray (Illumina, Inc., San Diego, CA, USA) by the Technology Centre, Institute for Molecular Medicine Finland (FIMM), University of Helsinki. This array covers more than 700,000 markers with an average spacing of 4 kb across the entire genome.

### DNA Sequencing

The *ARLTS1* variant status of the PCa patients used in Illumina genotyping was examined by direct sequencing. Sequencing was performed in an Applied Biosystems 3130xl Genetic Analyzer (Life Technologies Corporation, Carlsbad, CA, USA) according to the manufacturer's instructions. Primers and PCR conditions used in the mutation screening are available upon request.

### RNA Extraction and sequencing

Total RNA was extracted from 84 PCa cases and 15 healthy male relatives. All subjects belonged to the 31 Finnish HPC families mentioned above. Total RNA was purified from whole blood collected in PAXgene® Blood RNA Tubes (PreAnalytiX GmbH, Switzerland/Qiagen/BD) using the MagMAX™ for Stabilized Blood Tubes RNA Isolation Kit (Ambion®/Life Technologies, Carlsbad, CA, USA) and the PAXgene Blood miRNA Kit (PreAnalytiX GmbH, Switzerland/Qiagen/BD). The RNA quality was assessed using the Agilent 2100 Bioanalyzer and the Agilent RNA 6000 Nano Kit (Agilent Technologies, Santa Clara, CA, USA).

Library preparation, target enrichment and massively parallel paired-end sequencing of expressed transcripts was performed by Beijing Genomics Institute (BGI Hong Kong Co., Ltd., Tai Po, Hong Kong) using Illumina HiSeq2000 technology (Illumina Inc., San Diego, CA, USA).

### eQTL analysis

To obtain *ARLTS1* expression values, first, the reads from RNA sequencing were aligned with tophat2 using hg19 as the reference genome [15]. The raw read count for *ARLTS1* was calculated using HTseq, and read counts were transformed to normalised expression values using DESeq (www-huber.embl.de/users/anders/HTSeq/, [16]). The linear regression model implemented in PLINK was used to detect transcript specific variants. Associations in *cis* were delineated by a 1 Mb window upstream or downstream of the *ARLTS1* SNP rs9526582, as most of the *cis*-eQTLs are located within or close to the gene of interest [17]. In addition, we applied a new method based on probabilistic indices to test for eQTL. The new method is a non-parametric directional test (similar to the well-known Jonckheere-Terpstra test), implemented in our R-Package GeneticTools that is available on the Comprehensive R Archive Network (http://cran.r-project.org/package=GeneticTools), and a package description is under development (unpublished data). P-values were calculated using permutation tests and were adjusted for multiple testing by applying the Benjamini-Hochberg correction. We also calculated for each test size α in [0,0.1] the ratio of the amount of expected test rejections and the amount of observed rejections. The α for which the ratio of these two values was maximal, we chose also as an optimal test size.

### Gene expression dataset

All microarray gene expression data on cell lines (n = 1445) included in these analyses are publicly available via the Gene Expression Omnibus (GEO) (http://www.ncbi.nlm.nih.gov/geo/, accession numbers; GSE36133, GSE7127, GSE8332, GSE10843, GSE10890, GSE12777, GSE15455, GSE18773, GSE20126, GSE21654 and GSE24795) and GSK Cancer Cell Line Genomic Profiling Data (https://cabig.nci.nih.gov/tools/caArray_GSKdata) (2008) [18] (GlaxoSmithKline). The bulk of the gene expression data was acquired from the Cancer Cell Line Encyclopedia (CCLE) (GSE36133) [19]. We used samples from the most recent and widely cited Affymetrix microarray platform, HGU133_plus 2.0, to perform these analyses.

### Gene expression data Normalisation

Gene expression data normalisation was performed from the raw CEL files using the Aroma Affymetrix (Version 1.3.0) R package (http://www.aroma-project.org) based on custom CDF files (version 16) found at http://brainarray.mbni.med.umich.edu [20]. We processed expression for 19,003 distinct genes. All computations were performed in the R statistical environment, employing the BioConductor suite of packages.

#### Co-expression analysis of *ARLTS1*

The co-expression analysis method was described previously [10]. The meta cell line data (n = 1445) originated from over 48 human anatomical parts. A correlation value >0.30 and a p-value <0.05 were used as determinants for a statistically significant association. We performed multiple test corrections using Benjamini Hochberg and Bonferroni methods. We decided to use the Benjamini Hochberg (BH) method for selecting significantly co-expressed genes because it was moderately strict at calling a gene pair correlation a false positive.

### *In silico* functionality prediction

RegulomeDB (http://regulome.stanford.edu/) and HaploReg (http://www.broadinstitute.org/mammals/haploreg/haploreg.php) [21], [22] databases were used to further elucidate the role of eQTLs in gene regulation. RegulomeDB allows he features of DNA and regulatory elements of non-coding regions to be assessed, and HaploReg is a tool for developing mechanistic hypotheses of the impact of candidate regulatory non-coding variants on clinical phenotypes and normal variation.

### MDR analysis

The multifactor dimensionality reduction (MDR) program is publicly available from the internet (www.epistasis.org). We used version 2.0_beta_8.4 to examine gene-gene interactions. MDR detects interactions in relatively small sample sizes. MDR is a non-parametric, model-free data mining approach constructive induction algorithm that transforms the high-dimensional data into one-dimensional variables by pooling genotypes into high and low risk groups based on the ratio of cases to controls that have the genotype in question [23]. MDR selects one genetic model (a one, two, three or four stage locus) that most successfully predicts the phenotype or disease status, e.g., cancer. Data are then divided into ten equal parts to perform a 10-fold cross-validation. The model creates a training set (9/10 of the data) and testing set (1/10 data) to evaluate the prediction ability. The procedure repeats this protocol ten times and calculates the cross-validation consistency (CVC). CVC depicts the number of times that particular model is chosen as the best one of those ten intervals. From the MDR results section, testing balanced accuracy (TBA) shows how many instances are correctly classified. The *ARLTS1* genotypes from 102 PCa cases and 33 controls were combined with the GWAS data of 700,000 SNPs.

### Selection of genes and SNPs for MDR

The genes functioning in immune system processes and inflammation pathways were selected from a previously published comprehensive collection made by Loza MJ et al. [24] because MDR is not able to run data sets of thousands of SNPs within reasonable time limits. In short, the SNPs selected included those involved in apoptosis, cytokine signalling, Toll-like receptor signalling, leukocyte signalling, complement, adhesion and natural killer cell signalling. We also ran MDR with a subset of genes gathered from our previous *ARLTS1* co-expression studies (e.g., twelve genes within B-cell receptor [BCR] signalling pathway). The total amount of SNPs was 12,011 of which 4,764 were found in our Illumina Human OmniExpress GWAS data.

## Results

### RNA expression

**Figure 1:**
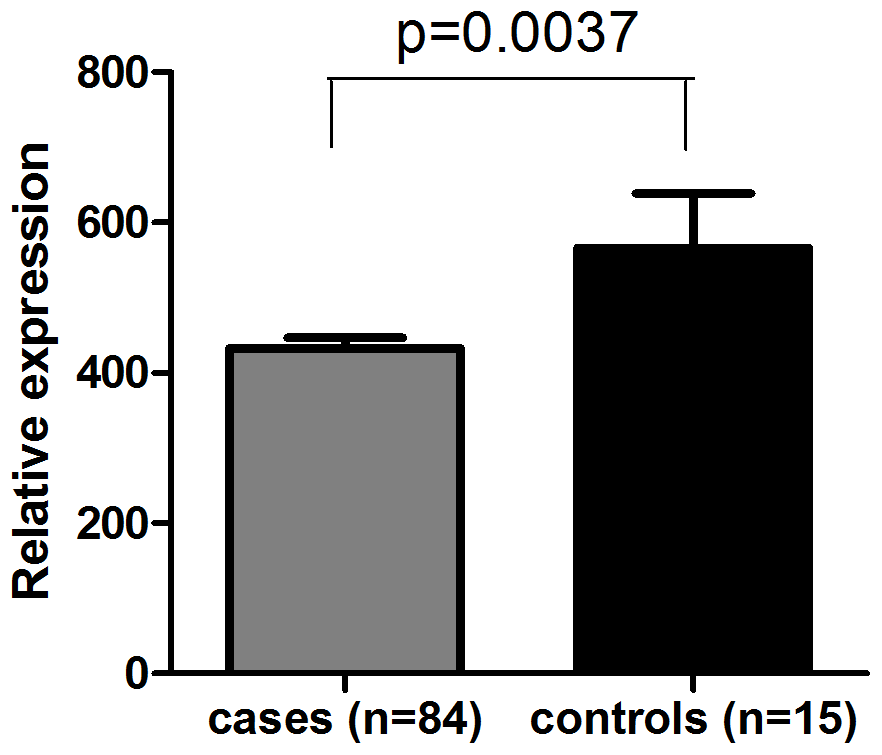
Relative *ARLTS1* RNA expression from PCa patients and healthy controls. Relative *ARLTS1* RNA expression was determined by RNA sequencing analysis. Columns represent means of individuals; bars represent SD.

The *ARLTS1* RNA expression levels were analysed from total RNA of 84 PCa cases and 15 controls. A significant decrease of the *ARLTS1* expression level in PCa cases was detected (p = 0.0037, Fig. 1). This is in concordance with our previous results from cell line, benign prostatic hyperplasia (BPH) and tumour specimen RNA expression data [10].

### eQTL analysis

**Figure 2:**
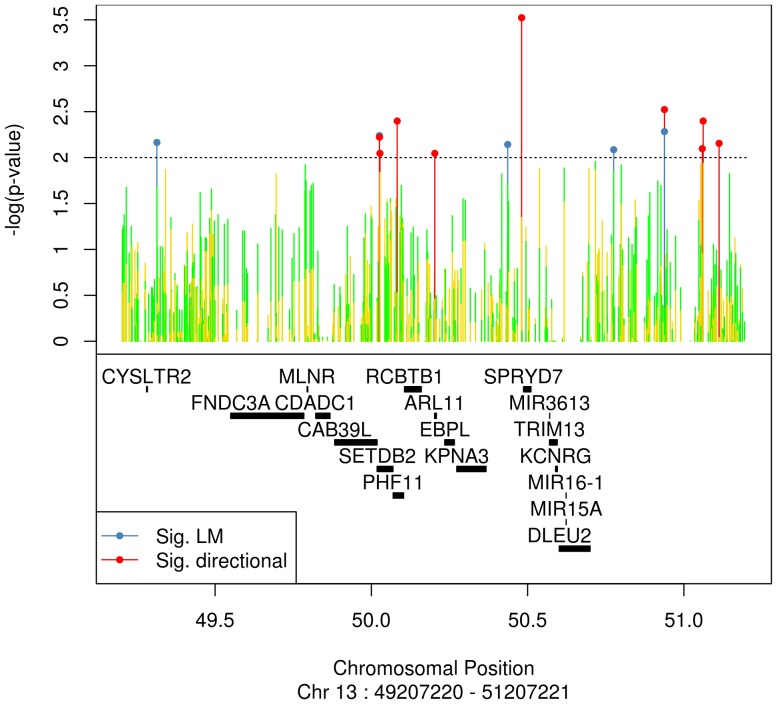
Schematic diagram showing the genomic locations of eSNPs gathered by eQTL analysis in PCa patients. The gene symbols are as follows: *CYSLTR2* (cysteinyl leukotriene receptor 2), *FNCD3A* (fibronectin type III domain containing 3A), *MLNR* (motilin receptor), *CDADC1* (cytidine and dCMP deaminase domain containing 1), *CAB39L* (calcium binding protein 39-like), *SETDB2* (SET domain, bifurcated 2/*CLLD8*), *PHF11* (PHD finger protein 11/NY-REN-34 antigen), *RCBTB1* (regulator of chromosome condensation [RCC1] and BTB [POZ] domain containing protein 1/*CLLD7*), *ARLTS1* (/*ARL11*, ADP-ribosylation factor-like 11), *EBPL* (emopamil binding protein-like), *KPNA3* (karyopherin alpha 3, importin alpha 4), *SPRYD7* (SPRY domain containing 7*/C13orf1*, chromosome open reading frame 1), *MIR3613* (microRNA 3613), *TRIM13* (tripartite motif containing 13), *KCNRG* (potassium channel regulator), *MIR-15A* and *MIR16-1* (microRNA genes 15a and 16-1) and *DLEU2* (deleted in lymphocytic leukemia 2).

To identify possible *cis*-acting genetic variants associated with *ARLTS1* transcript levels, we performed an eQTL analysis within a special area of the 13q14 region. By a linear regression model (PLINK), we were able to detect 5 eSNPs affecting *ARLTS1* expression (Table 2). When the calculation window was diminished from 1 Mb to 200 kb, only one SNP, rs7997377, remained. With the directional test performed by the R-Package analysis tool, 11 statistically significant eSNPs were found (Table 2), and two were in concordance with the PLINK linear regression model results. For both test procedures a test size of 0.01 was chosen. The eSNPs found by the directional test were located mainly in non-coding regions (Table 2). The genomic locations of the eSNPs found in the 13q14 region are visualised in Figure 2. Altogether, 468 genomic variants within the 1 Mb region originating from *ARLTS1* SNP rs9526582 were tested by a linear regression model and directional test.

**Table 2:** Chromosomal region 13q14 risk variants (eSNPs) associated with differential *ARLTS1* expression.

SNP	Gene	Position	Allele1	Allele2	P-value	Adjusted P-value
*Linear regression model*						
RS1886014	N/A	49321044	A	G	0,007	0,369
RS7997737	*SETDB2*	50033188	G	A	0,006	0,322
RS7337547	N/A	50443527	C	A	0,008	0,384
RS7995192	N/A	50782599	G	A	0,008	0,331
RS2532975	N/A	50945011	G	A	0,005	0,322
*Directional test*						
RS2075610	*MLNR*	49795705	G	A	0,010	0,322
RS7997737	*SETDB2*	50033188	G	A	0,008	0,322
RS1543513	*SETDB2*	50034684	A	C	0,007	0,322
RS9568232	*PHF11*	50089844	A	G	0,000	0,322
RS9562905	N/A	50210212	A	C	0,008	0,322
RS9568354	*SPRYD7*	50487993	A	G	0,002	0,000
RS2580189	N/A	50806640	A	G	0,009	0,322
RS2532975	N/A	50945011	G	A	0,001	0,322
RS1262781	N/A	51066171	A	G	0,010	0,322
RS1262774	N/A	51068896	A	G	0,006	0,322
RS17074618	N/A	51153475	A	G	0,006	0,322

After adjusting for multiple testing, a FDR of 39% in the linear model and approximately 32% in the directional test has to be accepted to keep the significant test results from the marginal p-values. For a more common FDR level of 10% one significant eSNP from the directional test remained significant (rs9568354). When we considered the ratio of expected and observed significant tests, a maximum ratio for α  =  0.011 in directional test was identified. For that α approximately 2.5 times more significant test results appeared than expected. Hence, we report also the above mentioned non-adjusted p-values for a significance level 0.01. With linear regression model, the amount of observed significant tests matched the amount of expected test rejections under the null hypothesis.

**Figure 3:**
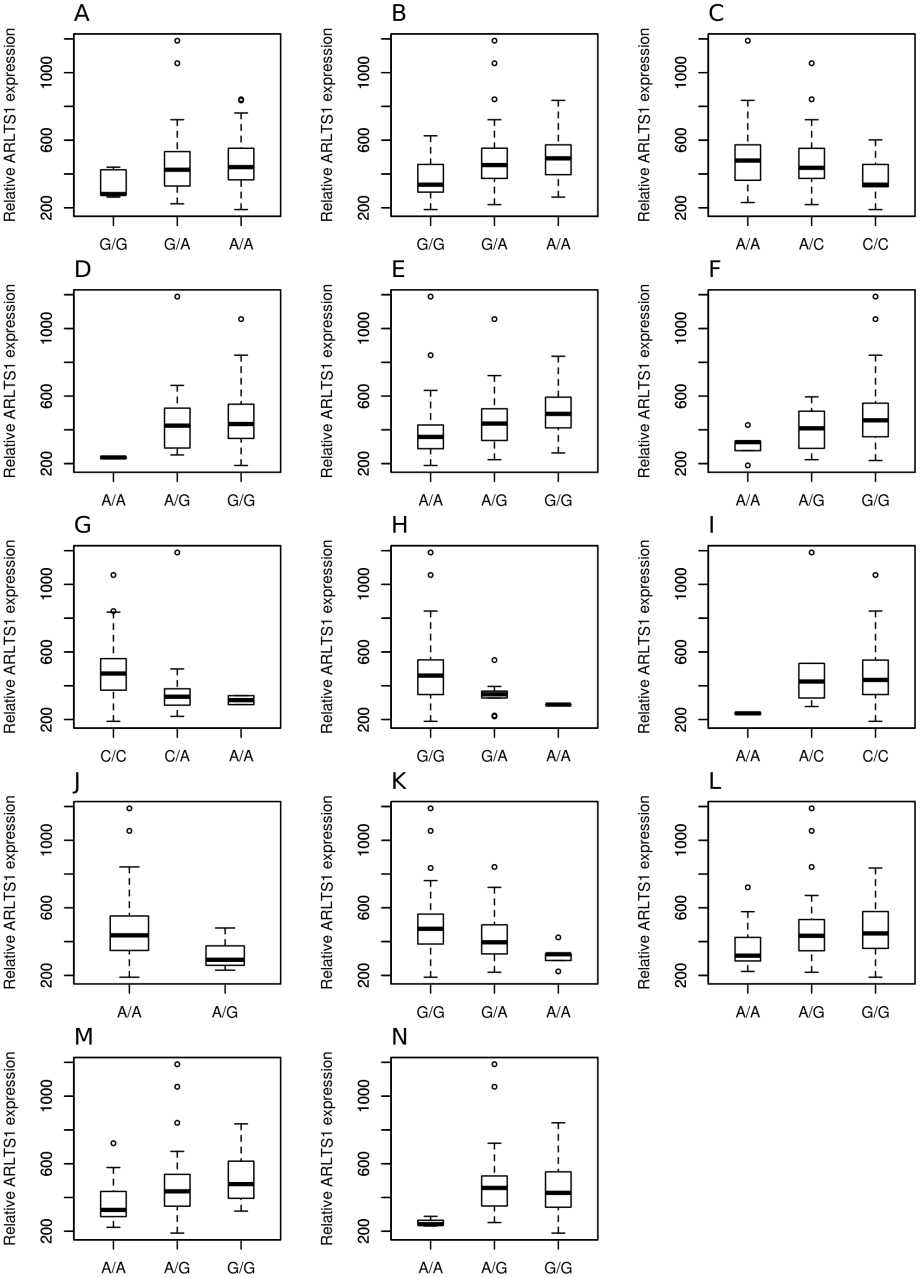
Correlation of the different genotype groups of eSNPs to *ARLTS1* RNA expression levels. A, rs2075610; B, rs7997737; C, rs1543513; D, rs9568232; E, rs9568354; F, rs1886014; G, rs7337547; H, rs7995192; I, rs9562905; J, rs2580189; K, rs2532975; L, rs1262781; M, rs1262774 and N, rs17074618.

The association of the eSNP genotypes with the *ARLTS1* expression level was calculated. A statistically significant correlation was naturally observed between all the 14 SNPs and *ARLTS1* transcript levels. The eSNP genotype - *ARLTS1* expression association box plots are depicted in Figure 3.

The functionality and possible transcriptional regulatory effect of the 14 eSNPs within genes found by eQTL was evaluated using ENCODE-data in the RegulomeDB and HaploReg databases. Altogether nine of the fourteen eQTLs are reported in RegulomeDB. The findings in the GM12878 lymphoblastoid cell line are emphasized below because this cell line resembles the tissue type from which the RNA sequencing data was retrieved.

The most substantial evidence for the regulation of *ARLTS1* was found for SNP rs2532975. Its regulatory role is supported by its location in a regulatory active region. According to the Chip-Seq data from the GM12878 cell line, rs2532975 resides in a BATF (basic leucine zipper transcription factor) binding site. In addition, using position weight matrix (PWM) matching, a CDC5 (cell cycle serine/threonine-protein kinase) binding motif has been identified that spans the genomic position of this variant. Furthermore, a promyelocytic leukemia zinc finger (PLZF) motif is reported in HaploReg. As reported by HaploReg, CDC5 binding efficiency decreases while PLZF binding increases. Additionally, the chromatin state in the region surrounding rs2532975 might adopt weak enhancer characteristics. This prediction is further strengthened by the presence of two histone marks in this region, H3k4me1 and H3k4me2, identified in the GM12878 cells.

Another possible candidate for *ARLTS1* regulation is rs9562905. In a Chip-Seq study, a POLA2 binding site was identified in a human embryonic stem cell line, H1-hESC, in the region surrounding rs9562905. HaploReg predicts active enhancer characteristics in the chromatin surrounding rs9562905 in the lymphoblastoid GM12878 cells, similar to rs2532975. This interpretation is supported by the presence of two enhancer associated histone marks, H3k4me1 and H3k4me2. In addition, a FAIRE-sequencing study conducted for the GM12878 cells also implies that this region is in an open chromatin state and is therefore likely to have regulatory activity.

The variants rs1543513, rs7997737 and rs9568354 share similar chromatin structural features in the GM12878 cells. According to HaploReg, the chromatin state associates with the weakly transcribed region. This prediction is confirmed by the elongation of histone mark H3k36me3 in the surrounding regions of the variants rs1543513, rs7997737 and rs9568354. According to RegulomeDB, rs1543513 is located within the Irx3 and Irx6 motifs. However, in HaploReg, the presence of these motifs is not reported. Similarly, the transcription factor binding motif of AIRE (autoimmune regulator) is reported for rs1262781 in RegulomeDB but not in HaploReg. A MZF1 (myeloid zinc finger 1) motif surrounding rs7997737 is reported in both databases. HaploReg reports a negative LOD score difference for rs7997737, which can be interpreted as lowered binding efficiency of MZF. Compared to the three variants mentioned above, rs9568232 shares similar characteristics regarding its chromatin state. According to HaploReg, this chromatin state is related to elongated transcription.

The variants rs2075610 and rs1262774 are located within regions most likely under the control of epigenetic regulation by the polycomb-group proteins in the GM12878 cells. In addition, DNase-Seq studies performed for several cell lines indicate an open chromatin state surrounding rs2075610. However, two repressed state chromatin histone marks, H3k27me3 and H3k9me3, have also been identified. Variant rs1262774 is not reported in the RegulomeDB.

The remaining variants are located in heterochromatin regions, according to HaploReg. For rs7995192, rs2580189 and rs1262781, this prediction is further confirmed by the presence of H3k27me3. In addition, another repressive state associated histone mark, namely H3k9me3, is present in the rs7995192 and rs2580189 regions. Although there is evidence of repressed state chromatin, RegulomeDB reports that transcription factor binding motifs do in fact span the genomic positions of rs2580189 and rs1262781.

Two motifs for XBP-1 (X-box binding protein 1) and ATF6 (activating transcription factor 6) span the region of rs1262781, according to RegulomeDB. HaploReg confirms the presence of XBP-1 and ATF6 motifs and an additional motif for SOX-17 (SRY [sex determining region Y] box 17). HaploReg reports lower predicted binding efficiencies for XBP-1 and ATF6 and a slightly increased binding efficiency for SOX-17.

RegulomeDB reports a ZNF143 (zinc finger protein 143) motif in the region surrounding SNP rs2580189. However, HaploReg does not report the presence of this motif. Taken together, the results gathered from RegulomeDB and HaploReg indicate that the *ARLTS1* eQTLs are located within regulatory areas of the 13q14 region.

### Co-expression analysis

The GeneSapiens mRNA expression database data, including *ARLTS1* expression, from 1445 cell lines (48 cancer subtypes) and prostate cancer tumours was available for interaction studies. Altogether 1381 genes with correlation value >0.30 and p-value <0.05 was found to be positively correlating with the *ARLTS1* gene. Using DAVID GO functional clustering, (http://david.abcc.ncifcrf.gov/tools.jsp) [25], [26] a strong association with nucleus and zinc-finger protein processes was illustrated when all the genes positively correlating with *ARLTS1* expression (n = 36) in the PCa cell line cohort were taken into account (with a more stringent correlation value >0.50). The group of nuclear processes (nucleus, intracellular organelles, transcription and DNA-binding) was enriched when data of PCa cell lines was studied. The enrichment score was 13.49 with a p-value 1.1E-32. The adjusted Benjamin score was 5.1E-30, and the cluster of zinc-finger binding proteins revealed a p-value of 9.0E-22. The same phenomenon was observed within the whole data of cell lines (meta cohort) with genes showing a correlation value >0.30. *ARLTS1* co-expression genes (with correlation value >0.50) from the meta cell line data revealed a category of immune system processes (B-/T-cell activation, leukocyte/lymphocyte differentiation and activation), with an enrichment score of 2.94 (p-value 5.57E-7, adjusted Benjamin score 2.9E-5).

*ARLTS1* co-expression data of genes negatively correlated to *ARLTS1* identified a strong gene ontology of glycoprotein and plasma membrane protein genes in PCa cell lines (n = 2722, correlation value<−0.50, enrichment score 52.13, p-value 3.4E-72 and 38.35, p-value 7.9E-32, respectively). Within the negatively correlating genes, a cluster of immunoglobulin domain containing proteins harboured an enrichment score of 12.23 with a p-value of 5.4E-24. The GO term “cytokine activity” revealed an enrichment score of 10.43 with a p-value of 6.5E-10 within negatively correlating genes. In addition to the result of *ARLTS1* negatively correlating genes, we also identified clusters of G-protein coupled receptors and cell-cell signalling.

Top five positively and negatively *ARLTS1* correlating genes in cell line data are presented in Table 3 (upper panel). Additionally, results of the *ARLTS1* co-expression with genes (*SETDB2, PHF11, SPRYD7, MLNR*) that harbored eSNPs are presented in the lower part of the Table 3, from the co-expression analysis performed in the meta cell line, prostate cell line data and prostate tumor data.

**Table 3:** *ARLTS1* Co-expression signatures from tumor specimens and cell lines.

Gene	Correlation value	P-value	Samples (n)	pval_corrected*	pval_corrected#
***Top five genes***					
*Co-expression in meta cell line data*					
*Positive correlation*					
*BTK*	0,69	0	2818	0	0
*GPR18*	0,66	0	2818	0	0
*CXorf21*	0,66	0	2818	0	0
*P2RY8*	0,66	0	2818	0	0
*PIK3CG*	0,65	0	2818	0	0
*Negative correlation*					
*NCKAP1*	−0,62	1,69E-295	2818	3,22E-291	7,70E-295
*CDC42BPB*	−0,59	1,64E-263	2818	3,11E-259	7,43E-263
*GIPC1*	−0,57	1,10E-239	2818	2,10E-235	5,01E-239
*PTMS*	−0,53	2,68E-208	2818	5,09E-204	1,22E-207
*KIAA0284*	−0,53	1,04E-203	2818	1,97E-199	4,71E-203
***Genes harboring eSNPs***					
*Co-expression in meta cell line data*					
*MLNR*	−0,15	1,35E-16	2818	2,57E-12	2,96E-16
***PHF11***	**0,44**	**0,000**	**2818**	**0,000**	**0,000**
***SETDB2***	**0,64**	**0,000**	**2818**	**0,000**	**0,000**
*SPRYD7*	0,28	0,000	2818	0,000	0,000
*Co-expression in PCa cell lines*					
***MLNR***	**−0,35**	**0,0352**	**36**	**1**	**0,081**
*PHF11*	0,09	0,608	36	1	0,704
***SETDB2***	**0,61**	**9,01E**-**05**	**36**	**1**	**0,001**
*SPRYD7*	0,17	0,336	36	1	0,451
*Co-expression in prostate tumor samples*					
*MLNR*	0,03	0,821	75	1	0,934
*PHF11*	−0,07	0,576	75	1	0,806
*SETDB2*	−0,07	0,547	75	1	0,788
***SPRYD7***	**−0,34**	**0,003**	**75**	**1**	**0,045**

* Bonferroni correction.

# Benjamini Hohchberg multiple testing correction.

The expression of *ARLTS1* was positively correlated with the expression of *SETBD2*, in both the whole data of cell lines (meta cell line cohort) (correlation value 0.64, p-value 0.000) and in the specific PCa cell lines (correlation value 0.61, p-value 0.00009) (Table 3). Expression of the *PHF11* gene was positively correlated with *ARLTS1* expression in the meta cell line data (correlation value 0.44, p-value 0.000). Expression of *MLNR* and *SPRYD7* was negatively correlated with *ARLTS1* expression. *ARLTS1* and *MLNR* had a correlation value of −0.35 (p-value 0.04) in PCa cell lines, and *ARLTS1* and *SPRYD7* had a correlation value of −0.34 (p-value 0.003) in prostate tumour specimens (Table 3). The strong negative correlation of *ARLTS1* and *SPRYD7* expression levels was also validated in our transcriptome data of 84 PCa cases and 15 controls.

### MDR analysis

By direct sequencing, we were able to detect six *ARLTS1* variants at the same amplicon from PCa patients included in the Illumina genotyping (n = 135). All the variants were previously known (rs117251022, rs3803186, rs147120792, rs3803185, rs138452698 and G446A [Trp149Stop]). To elucidate the genotypic *ARLTS1* interactions we used multifactor dimensionality reduction (MDR). Gene-gene interaction status between *ARLTS1* and genes functioning in immune system processes within 102 PCa cases and 33 controls was calculated, but we were not able to find any statistically significant *ARLTS1* interactions in this study cohort (data not shown).

## Discussion

*ARLTS1* is a cancer-predisposing gene with proven tumour suppressor properties. However, very little evidence on function, especially on pathways, is currently available. In this study, we were able to verify downregulated *ARLTS1* expression in the blood-derived RNA of PCa patient samples, demonstrating for the first time the effect of germline alteration on *ARLTS1* expression levels. Tumour suppressor function of the *ARLTS1* gene has been previously proven by Calin GA et al., who found that transduction of full-length *ARLTS1* to A549 cells in Nu/Nu mice decreased tumour growth when compared to empty vector [1]. The ability of *ARLTS1* to suppress tumour formation in preclinical models has also been observed with ovarian [27] and lung cancer cells [28]. However, these results are based on somatic mutations in cancerous cell lines, whereas our result reveals a novel expression difference at the germline level, supporting the role of *ARLTS1* as a tumour suppressor gene. In the future, these types of findings could be used to enable screening and detection of at-risk patients even before clinical diagnoses.

We have previously genotyped *ARLTS1* variants in prostate, breast and colorectal cancer [29] and produced a prostate cancer follow-up study [10]. In the first study [29], we reported a statistically significant association with *ARLTS1* variants T442C, G194T and prostate cancer risk. However, after adjusting for multiple testing, none of the results were significant. In the follow-up study with larger sample size, we reported a statistically significant association with T442C variant and prostate cancer risk [10]. We reported also a decreased or lost *ARLTS1* RNA or protein expression in clinical prostate tumors, prostate cancer cell lines and xenografts, supporting the role of *ARLTS*1 as a tumor suppressor gene. Thus, there is no conflict between the previous publications and this study that is confirming the tumor suppressor role of *ARLTS1*.

Here, 14 eSNPs located at the 13q14 region were shown to significantly influence *ARLTS1* transcript levels in PCa patients. The eQTL analysis was performed with two methods, a linear model approach and a directional test. One advantage of our directional test method over the commonly used linear model approach is its robustness against outliers and the weaker model assumptions. In the linear model approach, the expression values of the different genotype groups are considered to follow the same normal distribution up to a location shift parameter. If the location shift parameter is not equal to zero, it generates a testing problem. The directional test only assumes that the different expression values follow certain distribution functions, so a testing problem only occurs if the distributions of the genotype groups are stochastically ordered.

Interestingly, 5 of the eSNPs are located within the protein-coding genes *SETDB2*, *PHF11*, *SPRYD7* and *MLNR*. Two eQTLs were positioned in the *SETDB2* (SET domain, bifurcated 2) gene, also known as the *CLLD8* (chronic lymphocytic leukemia deletion region gene 8 protein) gene, which functions mainly in epigenetic regulation [30]. The *PHF11* gene, another hit for the eSNPs, is a positive regulator of Th1-type cytokine gene expression through nuclear factor kappa B, (NF-kB) [30], [31]. The third eSNP gene, *SPRYD7*, (SPRY domain containing 7/chromosome 13 open reading frame 1 *[C13or1]*) also known as chronic lymphocytic deletion region gene 6 protein, (*CLLD6*) [32], is proposed to have a tumour suppressor function because it has been detected to be downregulated in B-CLL patients [33]. The SPRY/B30.2 protein domain occurs in a variety of cellular proteins, mediates protein-protein interactions and negatively regulates cytokine activities [34], [35]. The fourth eSNP gene, *MLNR/MTLR1*, (G-protein-coupled receptor 38, GPR38) is a member of the G-protein coupled receptor 1 family and is identified as the motilin receptor [36]. The found eSNPs were not affecting the expression of the gene they resided in, suggesting the role of these eSNPs as specific regulators targeting *ARLTS1* expression. However, no explicit conclusions about the actual causal interaction between these variants and *ARLTS1* can be made without further functional validation. The eQTL result gathered in this study was from a relatively small sample set in which analyses encompassed 1 Mb up- and downstream of the *ARLTS1* gene. Thus, further validation in larger sample sets and genomic areas are warranted. However, in our results, one eSNP remained significant even after adjustments for multiple testing. Those adjustments are intricate in the case of eQTL analysis and studies with small sample sizes often suffer from a low detection rate after multiple testing adjustment [37]. One possible strategy to deal with this is to accept a relatively high FDR for the sake of later validation in a larger population. Here, no potential candidate eSNPs were excluded from further studies because the α with largest ratio between observed and rejected tests was considered as optimal test size.

Lymphoblastoid cell lines (LCLs) are widely used in eQTL studies because they are an easily accessible source of patient samples of a single cell type. Particularly with prostate cancer, the tumour specimens of multiple foci are hard to collect in large amounts making it difficult to reliably detect disease-associated traits. By using prostate cancer specimens, it is possible to find tissue-specific genetic effects, but the use of lymphoblastoid cell lines or whole blood of familial prostate cancer patients enables one to detect the possible heritable germline differences which may contribute to prostate cancer susceptibility. It has been argued that SNP-transcript approaches using LCLs of small sample sets are underpowered [38], but many studies have been able to find overlapping eQTLs in cell lines and primary tissues [39]–[41]. Our finding of the eSNP in the *SPRYD7* gene was endorsed in the co-expression data, in which the *ARLTS1* expression levels were significantly correlated with *SPRYD7* expression, and in the prostate tumour specimens (Table 3). Additionally, in the data from the ENCODE consortium, the significance and usage of LCLs are justified because the lymphoblastoid cell line (GM12878) is one of the three cell lines in the higher priority Tier 1 cohort [42].

There may be a joint effect between the 13q14 genes. For example, one larger transcript variant that consists of more than one gene has been proposed with *SETDB2* and *PHF11* [30]. Very little is known about the interactions in this area, particularly the genomic collaborators of the *ARLTS1* gene. A recent study identified cellular retinoic acid binding protein 2 (CRABP2) and phosphoglycerate mutase 1 (PGAM1) as novel ARLTS1-binding proteins using the in-frame cDNA library technique [43]. These proteins were not observed in the expression or genotype level of our data, so further studies are needed to elucidate the actual *ARLTS1* interacting genes and proteins.

Considering the interactions between *ARLTS1* and inflammation pathway genes, it was our hypothesis that *ARLTS1* would have interactions with inflammatory genes, as chronic inflammation has been proposed as a prostate cancer risk factor. With MDR analysis, we aimed to investigate the previously observed *ARLTS1* connection with immune system processes [10]. Another aim was to test the hypothesis of genes functioning in inflammation processes affecting prostate cancer risk. However, we failed to substantiate our hypothesis, and statistically significant association between *ARLTS1* T442C SNP and the inflammatory/immune system SNPs was not found within the 700,000 SNPs analysed. For the analysis, we extracted distinct SNP groups from 4,764 markers found from our data of 700,000 variants in total. One possible reason for the negative results may be the relatively limited sample set of 135 cases and controls because we were not able to generalise the results to the test data. We were also concerned about overfitting the data with MDR.

Our previously found association between *ARLTS1* T442C and prostate cancer risk may be caused by the interaction network of different SNPs in the 13q14 region. Variant T442C may be included in a very rare haploblock, or there may still be a missing variant because the region has been connected to other cancers in addition to PCa.

In conclusion, in this study we have shown that the *ARLTS1* expression level is influenced by changes in germline expression levels and that the *ARLTS1* gene is a quantitative trait locus for 14 eSNPs in the 13q14 region. Thus, our results indicate a more complicated network of changes that increase PCa risk than merely one genetic variant in *ARLTS1*.
